# Experimental Models of Autoimmune Hepatitis: Disease Fidelity and Translational Relevance

**DOI:** 10.1111/liv.70797

**Published:** 2026-07-14

**Authors:** Laura Elisa Buitrago‐Molina, Matthias Hardtke‐Wolenski

**Affiliations:** ^1^ Department of Gastroenterology, Hepatology, Infectious Disease and Endocrinology Hannover Medical School Hannover Germany; ^2^ Institute of Medical Microbiology University Hospital Essen, University Duisburg‐Essen Essen Germany

**Keywords:** animal models, autoimmune hepatitis, ConA, CYP2D6, fibrosis, FTCD, hepatic tolerance, murine model, translational immunology, Treg

## Abstract

Autoimmune hepatitis (AIH) remains difficult to study mechanistically in patients because disease initiation is rarely observed directly, the relevant autoantigens differ across subsets, and clinically meaningful outcomes such as chronic inflammation, fibrosis, relapse, and treatment response evolve over time. Animal models therefore remain indispensable. At the same time, the field has become increasingly heterogeneous. Acute immune‐mediated hepatitis systems, especially concanavalin A (ConA), dominate the recent literature because they are rapid, inexpensive, and experimentally tractable. However, ConA‐induced hepatitis is not an antigen‐driven autoimmune response and is better interpreted as acute bystander immune‐mediated liver injury than as a stand‐alone model of chronic AIH. In contrast, antigen‐driven adenoviral models based on cytochrome P450 2D6 (CYP2D6) or formiminotransferase cyclodeaminase (FTCD), as well as genetically predisposed or spontaneous tolerance‐defect models, provide stronger insight into loss of hepatic tolerance, chronicity, fibrosis, and the interaction between antigenic context and host susceptibility. This review proposes a pragmatic framework for evaluating AIH models on the basis of face validity, construct validity, predictive validity, chronicity, host susceptibility, and mechanistic fitness for a specific biological question. Using that framework, we classify current models into acute surrogate models, immunization‐ and xenoantigen‐based systems, adenoviral antigen‐driven chronic models, spontaneous and genetically predisposed models, transgenic or neoantigen‐driven tolerance models, and humanized or microbiota‐sensitive hybrid systems. We then synthesize what these systems have taught the field about central and peripheral tolerance, MHC and non‐MHC genetic susceptibility, sex‐ and age‐related disease context, CD4^+^ and CD8^+^ T‐cell biology, B‐cell function, regulatory T‐cell instability, impaired suppressive function, defective IL‐2‐dependent regulation, macrophage and innate lymphocyte participation, cell‐death programmes, and gut‐liver or liver‐microbiome interactions. A central conclusion emerges: AIH models are complementary tools with markedly different levels of disease fidelity, and they should not be treated as interchangeable. Acute systems remain useful for effector‐phase biology and first‐pass intervention studies, but the strongest translational inferences for chronic AIH come from antigen‐defined chronic models and selected tolerance‐defect systems. Future progress will depend on better benchmarking across models, stronger alignment with human immune profiling and ex vivo validation platforms, and wider use of tolerance‐restoring rather than purely anti‐inflammatory therapeutic strategies.

## Key Points

Animal models are essential for studying autoimmune hepatitis, but they do not all represent the same disease process. Acute models such as ConA‐induced hepatitis are useful for studying rapid immune liver injury, whereas chronic antigen‐driven and tolerance‐defect models are more informative for understanding long‐term autoimmune hepatitis, fibrosis, relapse, and treatment development. Choosing the right model for the right question is therefore critical for translating experimental findings into human disease.

## 1 Introduction

Autoimmune hepatitis (AIH) is a chronic immune‐mediated liver disease in which adaptive immunity is directed against hepatocellular antigens in a permissive inflammatory context. Clinically, the disease is defined by a combination of histologic hepatitis, serologic immune activation, characteristic but incomplete autoantibody profiles, and responsiveness to immunosuppression. Yet the causal sequence that leads from genetic susceptibility to chronic liver‐directed autoimmunity remains difficult to reconstruct in patients. Disease onset is seldom witnessed, the interval between initiation and diagnosis can be long, and liver tissue is usually sampled only after inflammation is already established. These features explain why animal models have remained central to the field from the earliest experimental liver‐protein immunization systems to current antigen‐defined and engineered tolerance‐restoration platforms [1, 2, 3].

The problem is not the lack of models, but the lack of a single model that reproduces the full human syndrome. Some systems are excellent for studying intrahepatic priming, cross‐presentation, and tolerance induction, but they terminate in deletion, ignorance, or transient inflammation rather than chronic AIH. Others reproduce sustained portal and lobular inflammation, lymphoplasmacytic infiltrates, and fibrosis, but only on selected genetic backgrounds and with defined non‐universal antigens. Still others are driven by global failures of immune regulation and are therefore invaluable for understanding tolerance breakdown, while at the same time being too pleiotropic to serve as straightforward disease surrogates. The rapid growth of recent ConA‐based intervention studies has reinforced this problem: many papers labelled as experimental ‘AIH therapy’ primarily interrogate acute immune‐mediated liver injury rather than chronic antigen‐driven autoimmune hepatitis.

This review therefore uses a deliberately functional perspective. Rather than ranking models by popularity or chronology, it asks three practical questions. First, what aspect of AIH does a given model actually reproduce? Second, what mechanistic or translational question is it best suited to answer? Third, where are the boundaries beyond which its conclusions should not be generalized? To keep the review useful, priority is given to landmark model‐establishing papers, model‐informative mechanistic studies, and representative therapeutic studies. The very large literature on phytotherapeutics and small molecules in acute ConA systems is discussed as a pattern rather than catalogued exhaustively.

## 2 What Should Count as a Relevant Animal Model of AIH?

A useful AIH model does not need to recapitulate every clinical feature of human disease. It does, however, need to declare which validity domain it addresses. Three domains are especially helpful. Face validity asks whether the phenotype resembles human AIH—for example, chronic portal and lobular inflammation, interface hepatitis, plasma‐cell rich infiltrates, hypergammaglobulinemia, or fibrosis. Construct validity asks whether the initiating mechanism is biologically meaningful—such as antigen‐specific immune activation, failure of central or peripheral tolerance, or a plausible interaction between genetic predisposition and inflammatory danger signals. Predictive validity asks whether interventions that matter clinically, especially corticosteroid‐based immunosuppression or tolerance‐restoring strategies, improve the phenotype in a way that is not trivially explained by non‐specific anti‐inflammatory effects [1, 2, 3].

Model relevance should also be judged against host and disease‐context variables that shape human AIH. These include MHC and non‐MHC genetic background, sex, age, and disease subtype. This is particularly important for type 2 AIH, which is enriched in the paediatric population, and for experimental systems in which susceptibility is influenced by genetic background and sex‐dependent regulatory mechanisms. In the type 2 AIH model, both MHC and non‐MHC genes contribute to disease susceptibility, and Foxp3+ regulatory T‐cell responses have been implicated in male resistance to experimental disease [4, 5]. These observations argue against evaluating models only by histologic inflammation or fibrosis. A model may reproduce liver injury but still miss critical determinants of autoimmune susceptibility.

From that perspective, a pragmatic AIH‐relevant model should satisfy most of the following criteria. It should involve liver‐directed adaptive immunity rather than sterile, toxic, or purely bystander inflammatory liver injury alone. It should show sustained or relapsing hepatitis rather than a single short‐lived inflammatory flare. Histology should matter at least as much as serum biochemistry because chronic murine liver inflammation can persist despite only modest transaminase changes, and a comparable dissociation is increasingly recognized in human AIH. The model should ideally permit assessment of fibrosis, recurrence, or treatment withdrawal. Most importantly, it should enable mechanistic interpretation: whether the decisive event is antigen encounter, failure of deletional tolerance, impaired Treg stability or suppressive function, defective IL‐2 regulation, abnormal B‐cell help, altered innate sensing, or a change in tissue context.

This framework also clarifies two recurrent interpretive errors. The first is to equate autoantibody formation with pathogenic AIH. Autoantibody responses are useful markers of breakdown of humoral tolerance and may contribute to disease through mechanisms such as antigen presentation, T‐cell help, and antibody‐dependent cytotoxicity. However, their presence alone does not necessarily prove liver‐damaging autoimmunity. In the FTCD/CYP2D6/SLA comparative work, humoral autoreactivity could be induced more broadly than chronic hepatitis, and induction of anti‐nuclear or anti‐SLA responses did not automatically translate into disease [6]. Similarly, recent work in spontaneous T‐cell‐driven emAIH suggests that autoantigen‐selected B cells may at times be bystanders rather than primary drivers [7]. The second error is to equate acute immune hepatitis with AIH. Acute systems can be extremely informative for effector biology, but they do not automatically model chronic autoimmunity, fibrosis, relapse, or antigen‐specific tolerance failure.

**FIGURE 1:**
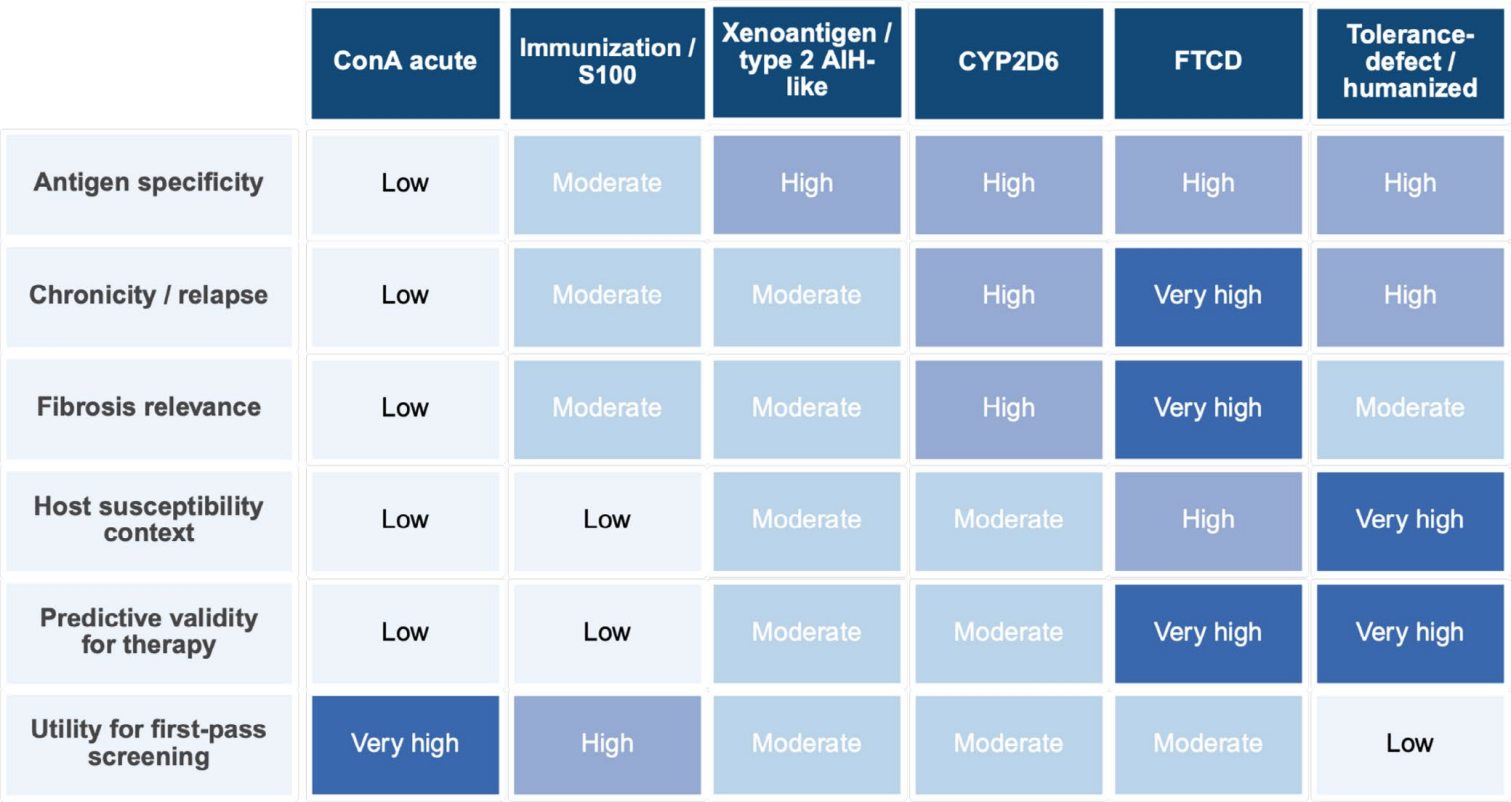
Benchmarking major AIH model classes across key domains of disease relevance. Acute models are useful for rapid screening, whereas antigen‐driven and tolerance‐defect models better capture chronicity, fibrosis, and translational relevance.

For practical use, it is helpful to think of AIH models as falling into three tiers. Tier 1 includes disease‐relevant chronic models, particularly antigen‐driven chronic systems and selected genetic tolerance‐defect models. Tier 2 includes partial disease models that reproduce one major axis of AIH, such as B‐cell help, microbiota dependence, or Treg dysfunction, or host susceptibility but not the full clinicopathologic syndrome. Tier 3 includes acute surrogate models, especially ConA, that are excellent for pathway dissection and first‐pass intervention screens but weak as stand‐alone representations of chronic AIH. The value of a study then depends less on the nominal model label and more on whether the model choice is aligned with the claim being made. These conceptual distinctions can be translated into a practical benchmarking framework across major AIH model classes (Figure 1), while the core characteristics of the major AIH animal model classes are summarized in Table 1.

**TABLE 1:** Core characteristics of the major AIH animal model classes.

Model class/example	Disease fidelity	Best use	Major limitation	Key references
Acute immune‐mediated hepatitis (ConA)	Acute T/NKT‐ and cytokine‐driven injury; poor stand‐alone fit for chronic AIH	Effector‐phase biology, rapid pathway dissection, first‐pass intervention screens	Not antigen‐driven; bystander immune hepatitis	[8, 9, 10]
Immunization/liver‐protein models (liver proteins, S100, liver homogenate)	Subacute to chronic depending on protocol; variable fibrosis	Historically important; useful for Th17/Treg, fibrosis, and cell‐death studies	Heterogeneous antigen source and weaker antigen‐specific construct validity	[11, 12, 13, 14]
Xenoantigen/type 2 AIH‐like models	Adaptive autoimmunity against human liver antigens; chronicity possible, host background influences susceptibility	Type 2 AIH concepts and human‐antigen immunization, genetic and sex‐dependent susceptibility	Phenotype depends heavily on host background and immunization design	[4, 5, 15, 16]
Adenoviral CYP2D6 model	Antigen‐specific chronic immune hepatitis with epitope spreading and fibrogenic potential	Molecular mimicry, type 2 AIH biology, disease modifiers such as NAFLD	Route, vector design, and strain strongly influence the phenotype	[17, 18, 19, 20]
Adenoviral FTCD/emAIH	Strongest disease‐fidelity chronic model with portal/lobular hepatitis and fibrosis	Tolerance breakdown, chronicity, intrahepatic induction and organ crosstalk, fibrosis, therapy benchmarking	Longer timeline; genetically restricted; antigen not universal across patients	[6, 21, 22]
Humanized susceptibility models (HLA‐DR4 NOD)	Chronic antigen‐driven disease with host susceptibility built in	MHC risk, host genetics, microbiota interactions	Breeding and interpretation are more complex	[23, 24]
Central tolerance‐defect models (Aire−/−, mTEC depletion, APS‐1‐related)	High construct validity for tolerance failure; often chronic or overlap‐like	Central tolerance, multiautoantigenicity, immune dysregulation	May resemble syndromic or overlap autoimmunity more than sporadic AIH	[25, 26, 27]
Peripheral regulation‐defect models (Tfh dysregulation, scurfy, DNase II‐deficient systems)	Fulminant or severe immune dysregulation phenotypes	Treg/Tfh circuits, innate sensing, inflammatory amplification	Extreme phenotypes can reduce generalizability	[28, 29, 30]
Transgenic/neoantigen tolerance systems	Usually transient, tolerogenic, or partial disease	Priming site, cross‐presentation, deletion, anergy, precursor‐frequency effects, restoration of immune homeostasis	Excellent mechanistic precision but poor fidelity for chronic AIH	[31, 32, 33, 34, 35]
Microbiota/infection hybrid systems	Usually modifier‐type models; can intensify chronic inflammation and fibrosis	Environmental triggers, gut‐liver/liver‐microbiome interactions, host‐microbe susceptibility	Often modify disease rather than fully generate it, some systems are not AIH‐specific	[14, 36, 37, 38, 39]

## 3 A Functional Classification of AIH Animal Models

### 3.1 Acute Immune‐Mediated Hepatitis Models

ConA is the dominant acute immune‐mediated hepatitis model in the contemporary literature. It is fast, inexpensive, and highly reproducible when dose, strain, and timing are controlled. Recent methodological work has further standardized its use, including a current protocol dedicated to ConA‐induced acute liver injury and explicit comparison of strain‐dependent sensitivity in BALB/c, C57BL/6J, and ICR mice [9, 10]. Mechanistically, the model is particularly valuable for studying rapid T‐cell activation, innate lymphocyte participation, cytokine cascades, endothelial and hepatocyte injury responses, and proof‐of‐concept anti‐inflammatory interventions. However, ConA‐induced hepatitis is not an antigen‐driven autoimmune response. It is better framed as an acute bystander immune‐mediated hepatitis induced by broad lectin‐driven immune stimulation, with prominent T‐cell and NKT‐cell activation.

This distinction is central for interpretation. ConA models a brisk, cytokine‐rich inflammatory insult and is therefore useful for dissecting acute effector circuits, including NKT‐cell regulation, macrophage polarization, dendritic‐cell responses, inflammasome activation, and hepatocyte death programmes [8, 40, 41]. It should not, however, be presented as a stand‐alone model of chronic AIH. It does not require a defined liver autoantigen, usually lacks the prolonged tempo of human disease, and only imperfectly models fibrosis unless it is modified or combined with additional interventions.

Accordingly, positive therapeutic results in ConA should be interpreted as evidence that a pathway or intervention modulates acute immune‐mediated liver injury, not as proof that the same intervention restores tolerance, prevents relapse, or treats chronic antigen‐specific AIH. For this reason, the term ‘immune‐mediated hepatitis’ or ‘bystander immune hepatitis’ is often more accurate than ‘autoimmune hepatitis’ when discussing isolated ConA data. We therefore consider ConA useful for first‐pass effector‐phase studies, but weak for translational claims about chronic AIH unless findings are validated in antigen‐driven or tolerance‐defect models.

### 3.2 Immunization‐ and Xenoantigen‐Based Models

The earliest experimental AIH systems relied on immunization with liver proteins and demonstrated a principle that remains foundational: adaptive immunity primed outside the liver can generate hepatotropic effector responses. Work from the 1980s showed transferability of hepatitis with sensitized splenocytes and established that cellular immunity could mediate target‐cell killing in liver‐protein models [11, 12]. These studies lacked the molecular precision now expected, but they framed AIH as an experimentally inducible autoimmune disease rather than an exclusively clinical syndrome.

A major conceptual advance came with xenoimmunization using human liver antigens. Lapierre and colleagues created a murine type 2 AIH model by immunization with human antigens, thereby moving from crude tissue homogenates toward antigen‐defined disease induction [15]. This line of work is particularly relevant because type 2 AIH has a strong paediatric association and because experimental susceptibility is shaped by host background, including MHC and non‐MHC genetic factors as well as sex‐dependent regulatory mechanisms [4, 5]. More recently, Thomas‐Dupont and co‐workers reported an additional type 2 AIH model built around a human liver protein, underscoring continued interest in antigen‐specific approaches beyond the classic FTCD and CYP2D6 platforms [16].

S100‐ and liver‐protein‐based models remain widely used because they are experimentally accessible and permit chronic or subchronic intervention studies. They have been especially useful for studying Th17/Treg balance, ferroptosis, fibrosis, microbiota effects, and cell‐based therapies. Representative recent work includes GPX4‐regulated ferroptosis in S100‐induced hepatitis, worsening of S100 hepatitis by dextran sulphate sodium‐induced dysbiosis, and modulation of S100 disease by mesenchymal stromal cells and all‐trans retinoic acid [13, 14, 42, 43]. Their main weakness is construct validity. Because the immunizing material is heterogeneous or incompletely representative of human autoantigenic targets, mechanistic conclusions about antigen specificity are inherently limited. These models should therefore be interpreted as useful systems for inducible immune‐mediated hepatitis and selected chronic readouts, but not as fully antigen‐resolved replicas of human AIH.

### 3.3 Adenoviral Antigen‐Driven Chronic Models

Adenoviral antigen‐driven models currently provide the strongest bridge between mechanistic tractability and disease fidelity. In the CYP2D6 system, transient viral delivery of a human liver autoantigen induces breakdown of immune tolerance and produces a type 2 AIH‐like syndrome with antigen‐specific immune responses, antibody formation, and, in some settings, fibrogenesis [17, 19, 44]. This platform also proved particularly informative for molecular mimicry, because T‐cell tolerance could be broken by similarity rather than strict identity, and for disease modifiers such as concomitant fatty liver disease, which worsens autoimmune hepatic injury in the CYP2D6 model [18, 20]. Importantly, earlier type 2 AIH work using the CYP2D6/FTCD antigenic system also demonstrated that disease susceptibility is not determined by antigen exposure alone, but by host genetic background, with both MHC and non‐MHC genes contributing to experimental susceptibility [4].

The FTCD‐based experimental murine AIH model (emAIH) extended this concept by coupling antigen specificity to a highly disease‐relevant chronic phenotype. In genetically susceptible NOD mice, adenoviral FTCD delivery induces chronic portal and lobular inflammation, interface activity, lymphoplasmacytic infiltrates, and progressive fibrosis in a CD4^+^ T‐cell‐driven process [21]. This model has two major advantages over most alternatives. First, it captures chronicity instead of a self‐limited flare. Second, it allows testing of standard immunosuppressive therapy, because corticosteroid‐based treatment improves histology, thereby supporting predictive validity. It therefore remains one of the strongest available models for asking how liver‐specific autoimmunity becomes chronic disease. Notably, these data also support the view that a preceding nonspecific inflammatory liver injury is not sufficient to explain disease development; rather, chronic AIH‐like pathology requires the convergence of antigenic context, danger signals, and a permissive host background [21, 22].

Subsequent comparative work clarified an equally important point: not all liver autoantigens are equivalent in their ability to generate disease. When FTCD, CYP2D6, and SLA/LP were compared across genetic backgrounds, breakdown of humoral immune tolerance was broader than induction of chronic hepatitis, and SLA/LP was particularly informative as a reminder that serologic immunogenicity does not automatically imply pathogenicity [6]. This work also made genetic context impossible to ignore. Autoantibody‐positive mice did not inevitably develop AIH, whereas Aire deficiency could render otherwise resistant backgrounds susceptible, linking antigenic exposure to host predisposition. Together with the earlier CYP2D6/FTCD type 2 model, these findings reinforce that antigen recognition, MHC restriction, non‐MHC susceptibility genes, and regulatory context jointly determine whether autoimmunity becomes chronic liver disease.

A related development was the introduction of humanized or susceptibility‐focused variants. The HLA‐DR4 non‐obese diabetic model and a broader humanized AIH mouse platform reinforced the importance of MHC context and connected host genetics to gut microbiota composition and liver inflammation [23, 24]. Together, these antigen‐driven chronic models support a concept that has become central to current AIH pathogenesis: disease requires more than antigen exposure. It emerges from the intersection of antigenic context, inflammatory danger signals, and a host background that fails to restore hepatic tolerance.

These models have also been especially valuable for questions of organ compartmentalization. Earlier work often emphasized the spleen or lymph nodes as induction sites, but experimental evidence from emAIH indicates that AIH induction can occur within the liver itself under appropriate conditions and that splenic influence depends strongly on timing [22]. Splenectomy before induction can exacerbate hepatic inflammation, whereas delayed splenectomy in established emAIH can induce biochemical remission and regenerative signatures [45, 46]. The lesson is not that one organ uniformly dominates, but that induction and maintenance of AIH are distributed processes shaped by time, antigen availability, and the balance between regulation and effector expansion.

### 3.4 Spontaneous and Genetically Predisposed Models

Genetic and spontaneous models are indispensable for understanding what happens when the immune system is biassed toward liver autoimmunity even before exogenous antigenic engineering. One influential line of work identified a fatal autoimmune hepatitis phenotype associated with dysregulated splenic follicular helper T‐cell generation, which was then mechanistically extended through studies of TNF‐α dependence, IL‐18‐producing dendritic cells, and hepatic CXCL9 expression [28, 47, 48]. These models are particularly informative for help‐dependent amplification loops and for the relation between secondary lymphoid tissue and hepatic injury. Their main value is therefore mechanistic: they reveal how systemic immune dysregulation can be focused onto the liver, but they should not be interpreted as simple replicas of sporadic adult AIH.

A second major axis involves defects in central tolerance. Bonito and colleagues showed that medullary thymic epithelial cell depletion can lead to autoimmune hepatitis, firmly linking failure of thymic negative selection to liver autoimmunity [25]. Related work from the APS‐1/Aire field demonstrated that AIH in this context is directed against multiple autoantigens rather than a single dominant target, and review work in the same space highlighted how the model broadens discussion from a specific liver antigen toward a systems‐level failure of tolerance architecture [26, 49]. These models have high construct validity for tolerance failure even when they do not mimic the average sporadic AIH patient. They are especially useful when the biological question concerns central tolerance, multiautoantigenicity, or the threshold at which impaired immune selection becomes organ‐directed liver disease.

Newer spontaneous or predisposition‐heavy systems extend this theme. Scurfy mice develop features of autoimmune liver disease overlap syndrome, highlighting the consequences of severe Treg deficiency [29]. dnTGFβRII Aire−/− mice develop an autoimmune hepatitis–primary biliary cholangitis overlap phenotype, emphasizing how combined regulatory defects can shift the disease away from classical isolated AIH [27]. DNase II‐deficient mice with IFNγ‐ and TLR9‐dependent hepatitis and the recently described *Helicobacter mastomyrinus* ‐triggered AIH model add further evidence that innate sensing and microbial exposure can act as disease initiators in a susceptible host [30, 39]. The main caveat across these systems is obvious: the closer a model comes to profound generalized dysregulation, the greater the risk that liver disease becomes one manifestation of broader autoimmunity rather than a selective AIH analogue. This caveat is important for translational interpretation, because treatment effects in such models may reflect correction of systemic immune dysregulation rather than disease‐specific restoration of hepatic tolerance.

### 3.5 Transgenic, Neoantigen, and Tolerance Models

Transgenic and neoantigen‐driven systems have made some of the most important conceptual contributions to liver immunology, even when they have not produced classical chronic AIH. Early work on intrahepatic CD8^+^ T‐cell activation showed how bone marrow‐derived cells, hepatocytes, and local antigen presentation shape the balance between productive immunity, bystander injury, and tolerance [31, 32, 33]. This literature helped establish a defining feature of the liver as an immune organ: antigen recognition in the hepatic environment often leads not to durable effector immunity, but to deletion, dysfunction, or regulation.

Subsequent studies refined this view. Liver sinusoidal endothelial cells can prime CD4^+^ T cells into suppressive populations, and liver‐specific CD8^+^ T cells can acquire cytotoxic potential only under very particular conditions [35, 50]. Even in inflammatory settings, the hepatic environment can remain strongly tolerogenic, a point reinforced by later work on tolerance induction during inflammation [51]. Consistent with this concept, administration of autoreactive T cells to healthy mice after local liver inflammation was not sufficient to sustain autoimmune liver disease; instead, hepatic immune homeostasis was restored through deletion of autoreactive T cells [34]. These models therefore excel when the question concerns precursor frequency, cross‐presentation, cell‐intrinsic dysfunction, or the site and context of priming.

Their limitation is disease fidelity. Because many transgenic systems employ unnaturally high precursor frequencies or engineered antigen expression, they can overestimate effector potency while underestimating the stochastic, polyclonal, and slowly amplifying nature of human AIH. The field should view them as mechanistic microscopes rather than clinical replicas.

### 3.6 Humanized and Emerging Hybrid Models

The most interesting current frontier lies in hybrid models that combine defined antigen systems with a second layer of biological realism. Humanized HLA‐driven systems already link host genetics to microbial and inflammatory context [23, 24]. Newer type 2 AIH models based on additional human liver proteins may diversify the experimental antigen space [16]. Infection‐ and microbiota‐sensitive systems likewise point toward a future in which environmental triggers are no longer treated as optional modifiers but as co‐determinants of disease induction [39].

Recent microbiota‐focused studies strengthen this concept. In a Tet2‐deficient setting, liver microbiome dysbiosis was shown to promote CD8^+^ Tc1‐mediated AIH‐like pathology through AhR‐linked microbial signals [38]. Although not a classical antigen‐defined AIH model, this system is important because it connects host epigenetic susceptibility, liver‐associated microbiota, and effector T‐cell differentiation in a disease‐relevant way. In a broader autoimmune context, translocation of *Enterococcus gallinarum* to the liver and systemic tissues can trigger autoimmune responses in genetically susceptible mice [36]. This work should not be overinterpreted as an AIH‐specific model, but it supports the general principle that microbial translocation to the liver can convert genetic predisposition into systemic and tissue‐directed autoimmunity.

A related development is the use of engineered delivery technologies not merely as therapies but as future modelling tools. Exosome‐modified AAV systems, targeted regulatory cell engineering, and increasingly precise in vivo gene‐delivery approaches may make it possible to combine antigen specificity, tissue restriction, temporal control, and immunoregulatory manipulation in a single platform [52, 53]. Such models are still emerging, but they point to where the field should be heading: from monolithic ‘AIH models’ toward modular experimental systems built around clearly stated biological questions.

## 4 What Animal Models Have Taught Us About AIH Pathogenesis

**FIGURE 2:**
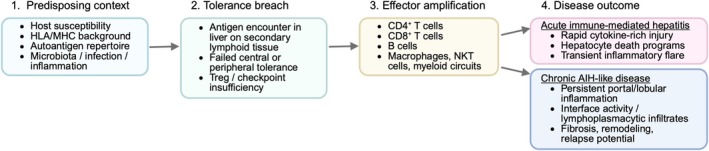
Modular framework of chronic AIH pathogenesis in vivo. Chronic disease emerges when host susceptibility, tolerance breach, and sustained effector amplification converge to produce persistent liver inflammation and remodelling.

The greatest value of animal models is not that they reproduce every patient phenotype, but that together they have revealed recurrent pathophysiological themes. The first is that hepatic tolerance fails through multiple, non‐equivalent routes. Central tolerance defects are demonstrated by mTEC depletion, Aire deficiency, and APS‐1‐related models [25, 26]. Peripheral tolerance failure is demonstrated by Treg instability, impaired suppressive function, defective IL‐2‐dependent regulation, abnormal Tfh/Tfr balance, and dysregulated checkpoint signalling [28, 54, 55, 56, 57]. Antigen‐driven chronic models add a third route: tolerance breakdown occurs when antigen is encountered in the right inflammatory context and in the right host, which is the central lesson of the FTCD/CYP2D6 work [6, 17, 21]. A second theme is the layered nature of effector immunity. CD4^+^ T cells are dominant drivers in emAIH and remain central in many immunization‐based systems, as underscored by work on CD4^+^ activation circuits and intrahepatic regulation [21, 58]. CD8^+^ T cells are particularly informative in transgenic and type 2 AIH‐related settings, and recent murine autoimmune liver disease work suggests that PD‐1^+^ CD8^+^ T cells can directly promote hepatocyte pyroptosis [59]. NKT cells, by contrast, are especially prominent in acute ConA‐mediated bystander hepatitis, where they help explain the speed and explosiveness of the model [8, 40, 41]. γδ T cells have emerged as additional pathogenic effectors in IL‐17‐skewed settings, linking metabolism to liver inflammation [60, 61]. Taken together, these observations support a modular view of chronic AIH pathogenesis, in which host susceptibility, tolerance breach, effector amplification, and disease outcome interact but are not equally represented across model systems (Figure 2).

The third theme is that B cells are neither uniformly dominant nor uniformly dispensable in AIH. B‐cell data across experimental systems are strongly context dependent. In xenoimmunized murine AIH, B‐cell depletion induced remission by reducing antigen presentation and T‐cell help, whereas in emAIH anti‐CD20 monotherapy altered the proteomic milieu without producing a clear histologic benefit [62, 63]. Consistent with this model dependence, splenic IL‐15‐producing B cells can amplify disease [64], whereas autoantigen‐selected B cells in spontaneous T‐cell‐driven emAIH may behave more as passengers than as initiators [7]. Recent work showing that extracellular inosine can promote B‐cell anergy and attenuate AIH further supports the view that the B‐cell compartment is biologically relevant, but its pathogenic role depends on immunologic context [65]. Thus, B cells should be viewed as context‐dependent contributors through antigen presentation, T‐cell help, cytokine production, and potentially antibody‐dependent effector mechanisms, rather than simply as markers of autoantibody positivity. Overall, these findings align well with earlier observations that breakdown of humoral tolerance may occur more readily than clinically meaningful autoimmune liver injury.

Innate and myeloid circuits have also become increasingly prominent. Bone marrow‐derived CD169^+^ macrophages can recruit CCR2^+^ monocytes and worsen AIH, while recent acute‐model studies continue to implicate macrophage polarization, efferocytosis, and innate sensor pathways in disease severity [66, 67]. The YTHDF2‐MDSC axis adds a separate regulatory layer by showing that myeloid suppressor networks can restrain disease [68]. These observations are important because they move AIH away from an overly T‐cell‐centric narrative and toward a multicellular network model.

A fourth theme is the growing attention to hepatocyte death programmes. Ferroptosis, pyroptosis, and, more recently, PANoptosis are now common mechanistic endpoints in the AIH literature. In chronic or semi‐chronic settings, ferroptosis has been implicated in S100‐induced disease and in recent studies of hepatocyte regulatory pathways, while pyroptotic injury has been linked to PD‐1^+^ CD8^+^ T‐cell activity and to microbiota‐driven fibrogenic inflammation [13, 59, 69, 70]. Acute bystander ConA systems have further expanded this space to include NLRP12‐driven PANoptosis [71]. These studies are mechanistically rich, but they should be interpreted with model awareness: a cell‐death programme validated only in acute ConA may not yet explain chronic AIH.

Finally, models have transformed understanding of organ crosstalk. The liver can be a site of priming, but it is not immunologically isolated. The spleen, gut microbiota, liver‐associated microbiota, microbial translocation, and systemic inflammatory tone all shape disease expression. Humanized and chronic models linked microbiota composition to liver inflammation, while faecal microbiota transfer, synbiotics, berberine, and microbiota‐derived tryptophan catabolites all modulate disease severity in experimental systems [23, 37, 72, 73, 74]. At the same time, DSS‐induced dysbiosis can aggravate S100 hepatitis, and microbial infection itself may be sufficient to trigger AIH‐like disease in susceptible settings [14, 39]. Recent microbiota‐sensitive and microbial‐translocation models further support the concept that host susceptibility can interact with liver‐associated microbial cues to promote AIH‐like or systemic autoimmune pathology [36, 38]. The gut‐liver axis is therefore best regarded not as an alternative explanation for AIH, but as a powerful modifier of antigen‐specific and tolerance‐dependent disease.

Across all these lessons, chronicity remains the decisive discriminator. A model that does not sustain disease may still explain activation, homing, or hepatocyte death, but it cannot on its own explain how AIH becomes a chronic fibrosing disease. This is why emAIH, the CYP2D6 platform, selected genetic tolerance‐defect models, and some immunization‐based chronic systems remain disproportionately important despite being less numerous than ConA studies.

## 5 Therapeutic Studies in AIH Models: How Much Translation Is Real?

Therapeutic interpretation in AIH depends first on model choice and only second on the intervention itself. The strongest translational inferences come from treatments tested in models that already possess reasonable disease fidelity. One of the major strengths of emAIH is that it responds to standard corticosteroid therapy, including budesonide‐ and prednisolone‐based immunosuppression, which gives the model unusually good predictive validity for preclinical AIH work [21]. By contrast, many treatment studies in acute ConA‐mediated bystander hepatitis convincingly reduce acute inflammatory injury without proving any effect on chronic tolerance breakdown, relapse prevention, or fibrosis.

Tolerance‐restoring strategies currently form the most conceptually attractive therapeutic class. Adoptive transfer of ex vivo expanded regulatory T cells restored peripheral tolerance in a murine AIH model more than a decade ago, and subsequent work showed that Treg‐specific IL‐2 therapy can re‐establish intrahepatic immune regulation [54, 75]. Recent studies extend this logic further upstream: serum amyloid A1 appears to destabilize intrahepatic Tregs, oestrogen receptor α inhibition can stabilize regulatory function, and CAR‐based in vivo Tfh reprogramming can restore tolerance in a mouse model of AIH [53, 56, 57]. Among newer interventions, these are the studies with the clearest mechanistic alignment to human AIH biology.

Cell‐based and vesicle‐based approaches form a second major therapeutic wave. Mesenchymal stromal cells, engineered PD‐L1‐ or PD‐L1/ICAM1‐enhanced stromal cells, MSC‐derived extracellular vesicles, and exosome‐modified AAV systems have all shown benefit in experimental hepatitis models [52, 76, 77, 78, 79]. These approaches are attractive because they combine immunomodulation with tissue‐protective effects, but most remain validated in acute or partially representative models. Their near‐term translational value will depend on whether they can be reproduced in chronic antigen‐driven systems.

Microbiota‐directed therapies deserve special attention because they now span both pathogenesis and intervention. Faecal microbiota transplantation, synbiotics, Bifidobacterium‐based interventions, berberine, and microbiota‐derived tryptophan catabolites all modulate disease severity in experimental AIH or immune‐mediated hepatitis, often by shifting the Treg/Th17 or Tfr/Tfh balance or by altering innate sensing pathways [37, 72, 73, 74]. Particularly noteworthy is the fact that not all such studies are confined to ConA: the Ad‐CYP2D6 model has also been used to explore intestinal flora and immune balance, which raises the translational confidence of this line of work [80].

A third category includes targeted pathway modulators. Hydroxychloroquine, zVAD, P2X4 deficiency, CEACAM1‐dependent IL‐2 regulation, AHRR silencing, and multiple macrophage‐ or T‐cell‐centred pathway interventions all show that immune hepatitis can be shifted by targeting specific signalling nodes [55, 68, 81, 82, 83, 84]. These studies are often mechanistically elegant, but their translational weight varies enormously depending on whether they were shown in chronic disease‐relevant models or only in acute surrogate systems.

The lowest tier of translational confidence is the vast literature of natural compounds and small molecules that has accumulated in single‐dose acute ConA experiments. This literature is not useless; it is often a valuable source of pathway hypotheses and sometimes a rapid screen for hepatoprotective or immunoregulatory effects. Yet as a class, it seems to be overinterpreted. A therapy that blunts acute ConA hepatitis may be anti‐inflammatory, anti‐oxidative, or cytoprotective without restoring tolerance or altering chronic AIH. The recent comprehensive review of natural products in AIH is therefore best read as a map of hypothesis generation, not as evidence that the field is close to replacing standard immunosuppression with nutraceuticals [85]. The practical hierarchy of therapeutic evidence across AIH model systems is summarized in Table 2.

**TABLE 2:** A practical hierarchy of therapeutic evidence across AIH model systems.

Therapeutic strategy	Representative platform(s)	What the data support	Translational confidence
Standard corticosteroids	Prednisolone and budesonide in emAIH	Benchmark for predictive validity and one of the strongest translational anchors in the field	High
Tolerance‐restoring cellular therapy	Adoptive Treg transfer	Proof that peripheral tolerance can be re‐established rather than merely inflammation suppressed	High
Targeted immunoregulation	Treg‐specific IL‐2	Supports a central role for defective intrahepatic regulation in disease maintenance	High
Engineered immune reprogramming	In vivo CAR‐Tfh reprogramming	A next‐generation circuit‐directed strategy with strong conceptual relevance	Promising/early
B‐cell‐directed modulation	Anti‐CD20; inosine‐induced B‐cell anergy	B‐cell function is important but context dependent	Intermediate
Cell‐based stromal therapy	MSCs; PD‐L1‐ or PD‐L1/ICAM1‐enhanced MSCs	Consistent anti‐inflammatory and immunoregulatory effects	Intermediate
Extracellular vesicles/gene delivery	MSC‐EVs; exosome‐modified AAV; exosome drug delivery	Technologically attractive and increasingly sophisticated platforms	Intermediate
Microbiota‐directed therapy	FMT, synbiotics, berberine, tryptophan catabolites	The gut‐liver axis is therapeutically tractable across multiple models	Intermediate
Pathway‐targeted small molecules	Hydroxychloroquine, P2X4 modulation, AHR/AHRR targeting, necroptosis/ferroptosis modulation	Useful for pathway validation	Intermediate to low unless confirmed in chronic models
Natural products in ConA	Large phytochemical and nutraceutical literature	Hypothesis‐generating and screen‐friendly but weak as stand‐alone chronic AIH evidence	Low

## 6 Major Limitations of the Current Field

The first major limitation is model imbalance. The current literature is heavily weighted toward ConA‐based bystander hepatitis and other acute immune‐injury systems, whereas comparatively few studies use antigen‐defined chronic models despite their much higher disease relevance. This imbalance distorts the field: pathways that are easy to detect in acute injury become overrepresented, while questions of chronicity, relapse, fibrosis, and tolerance restoration remain underpowered.

The second limitation is inconsistent language. Many studies label ConA‐induced bystander hepatitis or other acute systems as ‘autoimmune hepatitis’ without acknowledging that they primarily model acute immune‐mediated hepatitis. This terminological slippage matters because it affects how therapeutic claims are interpreted. The third limitation is insufficient standardization. Histologic scoring, timing of tissue collection, fibrosis assessment, sex stratification, and comparator therapies vary widely across studies. Head‐to‐head benchmarking between models is still uncommon, which makes it hard to know whether a given pathway is model‐specific or generalizable.

A fourth limitation is publication structure. Positive interventional studies, especially in the natural‐product space, are abundant; negative results and failed replication across models are much less visible. This is particularly problematic in AIH, where differences in strain, microbiota, route of antigen delivery, vector design, and timing can alter the phenotype profoundly. Finally, the field still underintegrates human immune data. The most sophisticated models will remain underexploited if they are not calibrated against patient‐derived information on autoantigen specificity, T‐cell state, tissue imprinting, and immune exhaustion.

## 7 Future Directions

The next generation of AIH modelling should move in three directions at once. First, the field needs consensus benchmarking. At minimum, model studies should state whether the system is acute or chronic, whether it uses a defined autoantigen, whether fibrosis occurs, whether histology was scored blindly, and whether a standard immunosuppressive comparator was included. Second, the field should prioritize combinatorial models that unite defined antigen systems with genetic susceptibility, relapse paradigms, fibrosis readouts, and environmental modifiers such as microbiota composition or infectious triggers.

Third, model refinement should increasingly be guided by human immunology. Single‐cell profiling of autoreactive CD4^+^ T cells in autoimmune liver disease, clonal analysis of SepSecS‐specific B and T cells, and broader evidence that autoantigen‐specific CD4^+^ T cells can persist in an exhausted state provide a much more granular blueprint for what relevant models should reproduce [86, 87, 88]. Recent immune profiling studies that link AIH to additional candidate antigens further support the idea that future models may need to move beyond the currently dominant antigen repertoire rather than merely recycle it [89].

Human ex vivo and organoid‐based platforms should become an additional calibration layer for AIH modelling. Precision‐cut liver slices, liver organoids, immune‐cell co‐cultures, and liver‐on‐chip or other microphysiological systems offer human‐cell specificity, controlled perturbation, and the possibility to interrogate hepatocyte–immune, endothelial–immune, and myeloid–parenchymal interactions without relying exclusively on mouse biology [90, 91]. Their main role, however, should be complementary rather than substitutive. Current ex vivo systems cannot yet fully reproduce systemic tolerance breakdown, lymphoid‐organ crosstalk, host genetics, microbiota‐dependent triggering, relapse, or the long‐term evolution of fibrosis. They should therefore be used to validate human relevance, test cell‐specific mechanisms, and benchmark therapeutic candidates emerging from in vivo models, rather than to replace chronic antigen‐driven or tolerance‐defect models altogether.

Technologically, the most promising path is toward antigen‐specific immune reprogramming rather than broader immunosuppression. CAR‐based Tfh reprogramming, AAV‐ and exosome‐enabled targeting strategies, and regulatory‐cell engineering all point in this direction [52, 53]. If these approaches can be validated in chronic antigen‐driven models and aligned with human immune signatures, the boundary between ‘modelling disease’ and ‘building therapy’ will become much more productive.

## 8 Conclusions

Animal models of autoimmune hepatitis are best understood as complementary tools with different levels of disease fidelity, not as interchangeable representations of the same disease. Acute ConA‐based bystander hepatitis systems remain useful for dissecting effector pathways, but they should not dominate translational interpretation. Chronic antigen‐driven systems and selected genetic tolerance‐defect models are far more informative for how hepatic tolerance fails, how fibrosis develops, and how durable remission might be restored. The central task for the field is therefore not to identify one perfect model, but to use each model with epistemic discipline, mechanistic clarity, and closer calibration to human disease.

## Author Contributions

Laura Elisa Buitrago‐Molina and Matthias Hardtke‐Wolenski conceptualized the review, reviewed and interpreted the literature, and contributed to manuscript writing and revision. Matthias Hardtke‐Wolenski supervised the work. Both authors approved the final version of the manuscript.

## Funding

The authors have nothing to report.

## Ethics Statement

Ethics approval was not required for this review article because no human participants, animals, or newly generated experimental data were involved.

## Consent

Patient consent was not required for this review article because no patient‐level data or identifiable patient information were used.

## Conflicts of Interest

The authors declare no conflicts of interest.

## Data Availability

Data sharing not applicable to this article as no datasets were generated or analysed during the current study.
